# Determination of Triacylglycerols by HTGC-FID as a Sensitive Tool for the Identification of Rapeseed and Olive Oil Adulteration

**DOI:** 10.3390/molecules25173881

**Published:** 2020-08-26

**Authors:** Ying Qian, Magdalena Rudzińska, Anna Grygier, Roman Przybylski

**Affiliations:** 1 Poznań University of Life Sciences, Poznań, Wojska Polskiego 28, 60-637 Poznań, Poland; magdar@up.poznan.pl (M.R.); ankaje@gmail.com (A.G.); 2 University of Lethbridge, 4401 University Drive West, Lethbridge, AB T1K 3M4, Canada; przybylski@uleth.ca

**Keywords:** triacylglycerols, gas chromatography, validation, identification, olive oil, rapeseed oil, adulteration

## Abstract

Triacylglycerols (TGs) are the most common compounds in food lipids, accounting for 95% of the weight of edible oils. The aim of this study was to scrutinize a procedure for quantitatively assessing possible adulteration of olive and rapeseed oil through GC-FID analysis of TGs. The recovery of TG standards ranged from 21% to 148%, and the relative response factor (RRF) ranged from 0.42 to 2.28. The limits of detection were in the range of 0.001 to 0.330 µg/mL, and the limits of quantitation from 0.001 to 1.000 µg/mL. The validated method was used to determine the TGs in olive oil (OO), refined rapeseed oil (RRO), and their blends. Eight TGs were detected in refined rapeseed oil, and 10 in olive oil. The addition of 1% of olive oil to rapeseed oil or vice versa can be detected using this method. Three triacylglycerols were pinpointed as indicators of adulteration of rapeseed oil with olive oil (PPO, PPL, PSO). The method described here can be used for controlling the quality of these oils.

## 1. Introduction

**Figure 1:**
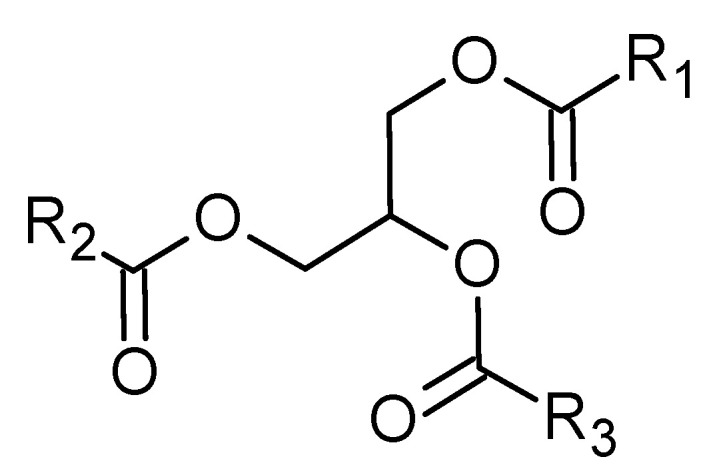
Chemical structure of triacylglycerol. R1, R2, R3–acyl moieties.

Triacylglycerols (TGs) are the most common compounds in food lipids, accounting for more than 95% of the weight of edible oils. The glycerol molecule is esterified with three fatty acids (Figure 1), although monoglycerols and diacylglycerols may also be present. The monoesters and diesters are often used in food applications as additives and emulsifiers. The TG composition of edible oils is rather difficult to analyze, due to its complexity, with vegetable oils containing a wide range of different fatty acids (FAs). However, such analysis is possible for mixtures of TGs with similar molecular weights but different molecular structures, due to the three possible positions of the fatty acid on the glycerol molecule.

A number of different analytical methods are used for TG determination. Gas chromatography (GC) is widely used due to its speed, convenience, and sensitivity [1]. High-temperature gas chromatography effectively separates TGs at temperatures above 300 °C [2]. Polyunsaturated fatty acids are prone to thermal degradation at this temperature, which can distort their true composition [1]. TGs have a high boiling point, and it is difficult to volatilize them [3]. For the purpose of high-temperature TG analysis, special columns have been developed to assist analysis at temperatures above 250 °C, to analyze diluted TG using GC directly [3].

A range of capillary columns has been used for the determination of triacylglycerols in edible fats and oils. A medium polarity open-tubular fused silica TG CB-type capillary column (Chrompack, São Paulo, Brazil) has been used to separate the TGs of several vegetable oils [4]. To analyze TG in cocoa butter, an equivalent 100%-dimethylpolysiloxane phase (DB-1) capillary column can be used [5]. The identification of milk fat in chocolate was performed on the basis of the TG composition determined using the CP-TAP CB columns (Agilent Technologies, Santa Clara, CA, USA) [6]. Lili et al. (2011) used (50%-phenyl)-methylpolysiloxane phase (DB-17ht) and 100%-dimethylpolysiloxane stationary phase (DB-1ht) capillary columns to quantify monoacylglycerols (MAG), diacylglycerols (DAG), and TGs in food lipids and oils. When the GC × GC-MS technique was used to determine TGs in fish oil, fused silica capillary columns (SLB-5ms) and ethylene glycol (Supelcowax-10) were used [7,8]. Adulterants of ghee were identified by analysis of triglycerides separated on a (5%-diphenyl)-dimethylpolysiloxan (CP-Ultimetal SimDist CP7532) column [9]. Supelco and Restek suggested using fused silica MET-Biodiesel or MXT-Biodiesel TG columns [10]. In addition, (5%-phenyl)-dimethylpolysi loxane (ZB-5ht) Phenomenex, (5%-phenyl)-methylpolysiloxane (DB-5ht, Agilent Technologies, Santa Clara, CA, USA), fused silica (Rtx-5SilMS, Restek, Bellefonte, PA, USA), and 5%-phenylmethyl polysiloxane (VF-5ms, Varian, Palo Alto, CA, USA) can be used to determine TGs [11]. The Capillary fused silica Rtx-65TG column by Restek is specially tested for triacylglycerols. The phase resolves TG by the degree of unsaturation, as well as by carbon number (www.restek.com). This column has been used for the determination of TGs in a range of dairy products [2].

Vegetable oils play an important role in the human diet. They are a source of essential fatty acids, vitamins, phytosterols, tocopherols, and other antioxidants. Global production of vegetable oils increased from 90.5 million tonnes in 2000–2001 to 207.5 million tonnes in 2019–2020. In 2018–2019, the consumption of palm, soybean, and rapeseed oils was respectively 70, 57, and 28 million tonnes [12]. In addition to traditional vegetable oils produced in large quantities, olive oil is also very popular, with a 2018–2019 world consumption of 3 million tonnes. The popularity of olive oil is associated with its high antioxidant content and its reputation as an element of a healthy Mediterranean diet. The main ingredients in olive oil are TGs, which can make up 98% of the content [4]. Olive oil has a high price, making it a target for adulteration with less expensive oils and fats. The problem of olive oil adulteration is not new but depends on the region or country where other cheap vegetable oils are produced [12]. It has been found that the adulteration of olive oil with rapeseed oil cannot be detected by measuring the refractive index, viscosity, or melting point [3]. However, triacylglycerol analysis by gas chromatography could be a rapid and sensitive method for identifying the adulteration of olive oil with rapeseed oil.

The aim of this study was to explore a method of quality and quantity analysis of TGs using GC-FID, and to employ this to detect the adulteration of olive oil by rapeseed oil.

## 2. Results and Discussion

### 2.1. Optimization and Validation of the GC-FID System

**Figure 2:**
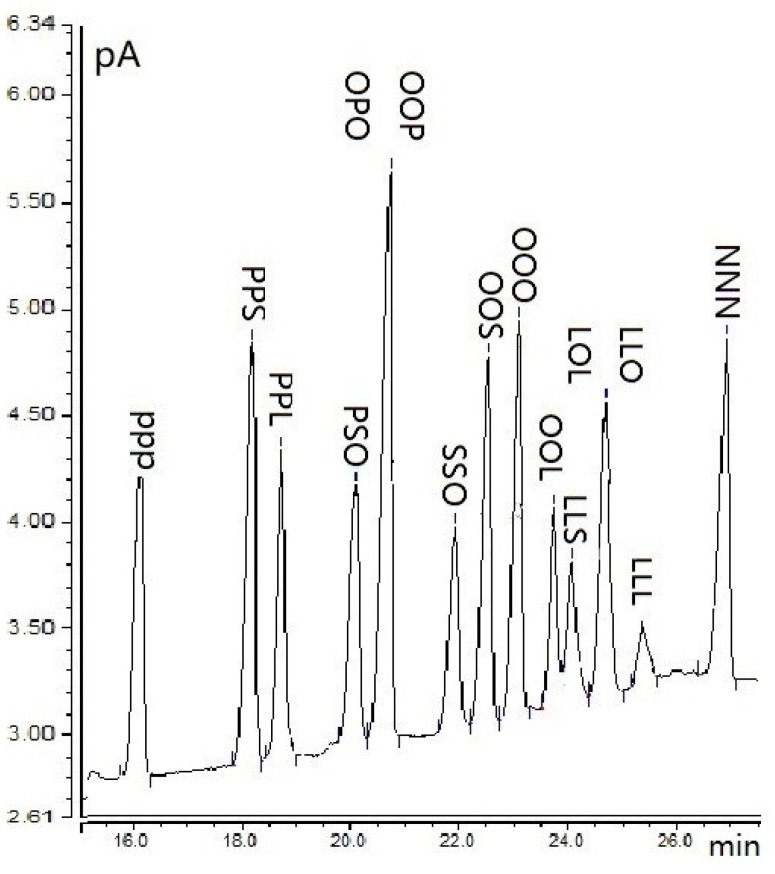
GC-FID chromatogram of triacylglycerols (TG) standards.

Basic data on the chemical structure of the analyzed TGs are presented in Table 1 and Figure 1. Figure 2 shows the chromatogram of the separated TGs. The carbon numbers of TG standards were used to optimize the separation, and ranged from 48 (PPP) to 57 (NNN); the double bond number was 0–6. The equivalent of carbon number (ECN) was calculated by the relation ECN = CN-2 × DB, where CN is a carbon number and DB is the number of double bonds [13]; this ranged from 42 to 57. The calculation of the equivalent carbon number is a powerful tool for identifying TG [14]. Table 1 presents TGs eluted from the column in order of increasing number of carbon atoms, and of increasing unsaturation for the same number of carbon atoms. In terms of the same CN, the elution order of TGs was increased by DB. PPP appeared first, due to having the lowest CN of 48. NNN, with CN 57, was an uncommon TG that eluted last. The peaks of TGs with the same CN and DB are very close, such as OOL and LLS, LLO, and OOLn. Two sets of TG positional isomers—OOP and OPO, and LLO and LOL—were eluted together. The relative retention times (rrt) were calculated using Equation (1) and ranged from 0.70 to 0.80 for three TGs, 0.80 to 0.90 also for three TGs, and more than 0.90 for 11 standards of TGs (Table 1).

For validation, the HTGC-FID method was used to determine the TGs in edible oils. To this end, solutions of 17 individual standards were prepared. Relative response factors (RRFs), recovery, limits of detection (LOD), limits of quantitation (LOQ), precision, and repeatability are presented in Table 1. The recovery of TG standards ranged from 21% to 148%, and RRF ranged from 0.42 to 2.28. The limits of detection were in the range of 0.001 to 0.330 µg/mL, while the limits of quantitation were in the range of 0.001 to 1.000 µg/mL. The precision of the HTGC-FID method was evaluated by assessing intraday and interday precision by analyzing TG solutions containing 1.25 µg/mL of each standard, reporting the retention times, and calculating the normalized areas. The intraday precision on the retention times of the TG was lower than 0.5%, and area precision was below 5% (data not showed). The interday precision on the retention times of the standards was less than 1.0%, and the peak area was less than 27%. In the reference, a wide range of precision was accepted, depending on the complexity of the matrix.

For the standards, solutions of TG calibration curves were prepared in the range of 0.5 to 10.0 mg/mL. Regression coefficients for standard curves ranged from 0.9900 for POO to 0.9980 for POP.

### 2.2. Identification of TGs in Olive Oil and Refined Rapeseed Oil

**Figure 3:**
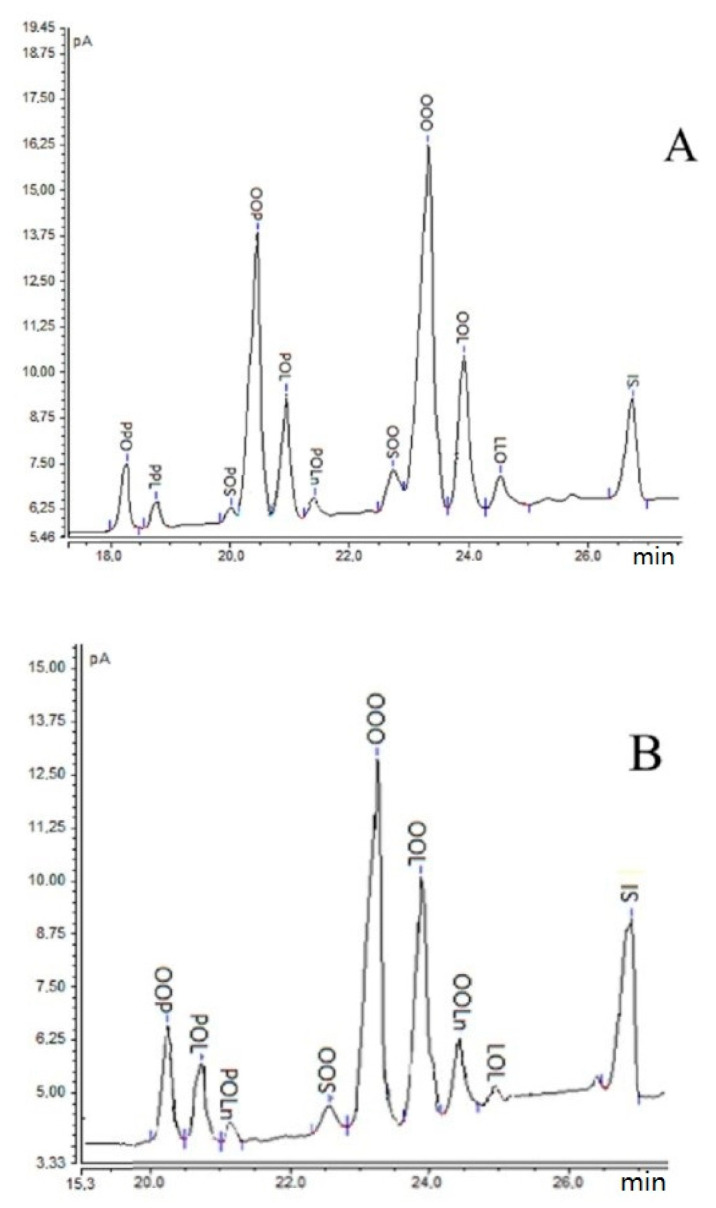
GC-FID chromatograms of TG in (**A**) olive oil (OO) and (**B**) refined rapeseed oil (RRO).

**Figure 4:**
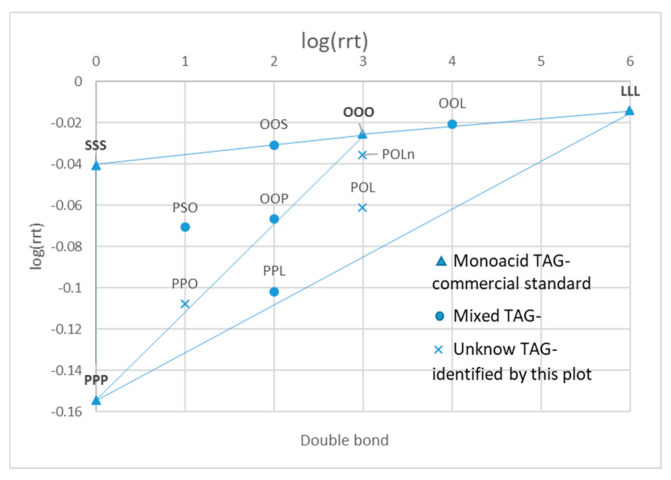
Plot for identification of unknown TGs in the tested refined rapeseed oil (RRO) and olive oil (OO).

The logarithms of standards’ relative retention times (rrt) were used to develop the plot used for identification [15,16] (Figure 3). Firstly, the saturated original standards were located on the graph on the double bonds number (DB) equals zero axis. Secondly, the monoacid original standards were positioned on the graph, and these points were connected by lines. Then, the TGs constituting of two types of fatty acids were located over the line connecting the two corresponding monoacid TGs. TGs with three different fatty acids were placed in the space between lines connecting the monoacid TGs. Following this, the points of unknown peaks were placed on the graph and identified (Figure 4).

### 2.3. Determination of TGs in Blended Olive Oil and Refined Rapeseed Oil

The validated method was used to determine the TGs in olive oil (OO), refined rapeseed oil (RRO), and their blends. The results are shown in Table 2, and the GC chromatograms of TG in olive oil and refined rapeseed oil are presented in Figure 3.

Eight TGs were detected in the refined rapeseed oil, and 10 in the olive oil (Table 2). The main TG in both oils was OOO, which was made up of 46% of RRO and 52% of OO, respectively. The next TGs in RRO was OOL (21%) and OOLn (13%), while OO contained OOP (25%) and OOL (9%). OOP, POL, POLn, LLO, and OOS were both detected in RRO (6%, 6%, 3%, 4%, 1.5%, respectively) and OO (25.4%, 6%, 2%, 1%, respectively). Three TGs (PPO, PPL, and PSO) were determined in OO (2%, 1%, 1%, respectively) but were not detected in RRO, and one TG OOLn determined in RRO (21%) was not found in OO. PPO made up 2% of olive oil, and PPL and PSO made up 1%. The amount of OOLn in RRO was 13%. These results agree with the literature data [4,14,17]. PPO, PPL, and PSO were detected in olive oil, palm oil, and peanut oil [4], but were not identified in rapeseed oil [14].

OOLn was identified in pine seed oil (8%) and soybean oil (2%), but was absent from olive, sunflower, sesame, corn, wheat germ, rice bran flax seed, melon, and pomegranate seed oil [4,16,18]. These TGs could serve good indicators for identifying blends of RRO and OO.

Most of the literature data on triacylglycerols in edible oils are based on their distribution by ECN, and describe the percentage of these groups [14,19,20]. The percentage composition of TGs depends on the number of compounds detected. In the tested RRO, triacylglycerols with ECN48 dominated (52%), followed by ECN46 (27%), ECN44 (20%), and ECN50 (1%). TGs with ECN48 were the main compounds in olive oil (79%), while ECN46, ECN50, and ECN44 made up 16%, 4%, and 1%. TGs with ECN48 made up 62%–72% of high oleic rapeseed oil (OOO + GLO), 43%–49% of medium oleic acid rapeseed oil (OOO + GLO), and 25%–27% of low oleic high erucic rapeseed oil (OPO + GLP + OOO + GLO + ErLnP + GGLn) [14]. The composition of extra-virgin olive oil ranged from 42% (OOP + OOO) to 50% of TGs with ECN48 [17,20].

The determination of individual TGs in rapeseed and olive oils allows the identification of key TGs that may be distinguishing features of adulterated oils. When 1% of OO was added to RRO, peaks of PPO, PPL, and PSO were detected at 0.8, 0.9 and 0.2 mg/g, (0.08%, 0.10%, and 0.01%), respectively (Table 2). The content of OOP increased from 47.4 mg/g in RRO (5%) to 71.5 mg/g (8%) in blended oil. When 2.5% OO was added to RRO, PPO content increased to 3.9 mg/g (0.44%), PPL to 1.9 mg/g (0.21%), PSO to 1.4 mg/g (0.16%), and OOP to 82.4 mg/g (9.24%). The contents of these TGs increased in proportion to the fraction of OO in RRO.

When RRO was added to OO, the level of OOLn was used as an indicator of adulteration. We observed an increase in the content of this TG in blends from 2.2 mg/g (1% RRO/OO) to 4.1 mg/g (2.5% RRO/OO), 6.0 mg/g (5% RRR/OO) to 12.2 mg/g (10% RRO/OO) (Table 2). Changes in the content of other TGs in the blended oils were not unambiguous, and can only suggest the adulteration of olive oil by rapeseed oil.

When OO was adulterated by soybean, sunflower, or corn oils, the absolute value of the differences between the theoretical and experimental ECN42 (LLL) contents was the most effective means of detecting even low levels of adulteration [20,21]. The content of linoleic acid in rapeseed oil is much lower than in soybean, sunflower, or corn oils, and LLL was not detected in our experiment. The greatest differences between the theoretical and experimental content of TG in the blended oils were detected for LLO and ranged from 3% to 9%.

## 3. Materials and Methods

### 3.1. Materials

All solvents of analytical grade were purchased from Sigma-Aldrich (Steinheim, Germany). Standards of the triacylglycerols 1,2-linoleoyl-3-oleoyl-sn-glycerol (OLL), 1,3-palmitoyl-2-oleoyl-sn-glycerol (POP), 1,3-palmitoyl-2-linoleoyl-sn-glycerol (PLP), trinonadecanoyl-glycerol (NNN), at over 99% purity, were obtained from Sigma-Aldrich (St. Louis, MO, USA). Trioleoyl-glycerol (OOO), 1,2-oleoyl-3-sn-palmitoyl-glycerol (OOP), 1,2-palmitoyl-3-linolein-sn-glycerol (PPL), 1,3-oleoyl-2-palmitoyl-sn-glycerol (OPO), 1,2-palmitoyl-3-stearoyl-sn-glycerol (PPS), 1,2-oleoyl-3-stearoyl-sn-glycerol (OOS), 1,2-stearoyl-3-oleoyl-sn-glycerol (SSO), 1-palmitoyl-2-stearoyl-3-oleoyl-sn-glycerol (PSO), 1,2-oleoyl-3-linoleoyl-sn-glycerol (OOL), 1,2-linoleoyl-3-oleoyl-sn-glycerol (LLO), 1,2-oleoyl-3-linolenoyl-sn-glycerol (OOLn), 1,3-linoleoyl-2-oleoyl-sn-glycerol (LOL), tristearoyl-glycerol (SSS), tripalmitoyl-glycerol (PPP), and 1,2-linoleoyl-3-steareroyl-sn-glycerol (LLS), at over 99% purity, were purchased from Larodan (Solna, Sweden). Refined olive pomace oil produced by Primadonna (Poland) and refined rapeseed oil produced by ZT Kruszwica (Warsaw, Poland) were purchased from a supermarket in Poland.

### 3.2. Sample Preparation

Solutions of 17 individual TG standards (PPP, PPS, PPL, PSO, OOP, OPO, SSO, OOS, OOO, OOL, LLS, LLO, LOL, SSS, OOLn, LLL, and NNN) were prepared. Briefly, 10 mg of each TG standard was dissolved in 10 mL of dichloromethane. Then 1 mL of each prepared solution was placed in a 2 mL autosampler vials, and 1 µL was injected and separated by gas chromatography.

We prepared 7 solutions of TG standards with concentration ranging from 0.05 to 10 mg/mL to determine linearity.

Before blending, the refined rapeseed oil (RRO) and olive oil (OO) underwent TG analysis. The blends consisted of 1%, 2.5%, 5%, and 10% additions of RRO to OO and OO to RRO. 30 mg of each oil (RRO, OO, and the blends) was weighed out in 10 mL volumetric flasks, and 3 mg of trinonadecanoyl-glycerol (NNN) was added to all samples as an internal standard (IS). The oils were then dissolved in 10 mL dichloromethane, and 1 mL of the solution was placed in 2 mL vials. All samples were prepared in triplicate.

### 3.3. HTGC-FID Conditions

Solutions of standards and oils were injected by autosampler (Thermo Scientific AI 1310) with a split ratio of 1:30 into a Thermo Scientific Trace 1300 gas chromatograph equipped with an RTX-65TG capillary column (30 m × 0.25 mm i.d. 0.1 μm; Restek Corp., Bellefonte, PA, USA) and flame ionization detector. The GC oven temperature was programmed to rise from 250 °C to 360 °C at 4 °C/min, before being held for 25 min. The injection port was held at 360 °C. Hydrogen (99.99%) was used as the carrier gas at a flow rate of 1.5 mL/min.

### 3.4. Validation

The parameters used to validate the method were: Fitting an analytical curve and determining its linearity, recovery, limit of detection (LOD), limit of quantitation (LOQ), precision, relative standard deviation (RSD), and repeatability. These parameters were calculated by peak area and evaluated according to AOCS Official Methods Ce 5b-89 (2009) and Cd 11c-93 (2009) [15,22].

**For linearity**, 7 solutions of each TG standard in the range of 0.05 to 10.0 mg/mL were prepared and injected 3 times per concentration level. The linearity of the method for each TG was evaluated by determining the coefficient (r^2^) after the construction of the analytical curves. The precision of the method was determined as the relative standard deviation from 6 replicates of the prepared standard mixture at 0.05 mg/mL concentration.

**The repeatability** of the method was evaluated to determine the content of TG in the mix of standards, using trinonadecanoyl-glycerol (NNN) as the internal standard (IS). The procedure was carried out on different days to obtain the intermediate precision. The relative standard deviation (RSD) was calculated according to equation RSD (%) = Standard deviation/Mean × 100%.

**Spike recovery** was calculated by using 10 mg of each commercial standard mixed in 1 mL dichloromethane. NNN was added as an internal standard to the calculate peak area of each standard. The recovery (%) = Final amount detected/Amount spiked × 100%.

**Limits of detection (LOD) and limits of quantitation (LOQ)** were calculated for each TG using 17 standards at the lowest concentration of 0.05 mg/mL. LOD was estimated as the ratio of blank signal to TG standard signal at the lowest concentration that was reliably distinguished from the blank sample and for which detection was feasible. LOQ was the lowest concentration of the compound for which quantitation was acceptable. LOQ was calculated for all TG standards at 3 times the limit of detection obtained for each TG.

### 3.5. Calculation of Relative Response Factors (RRF)

Trinonadecanoyl-glycerol (NNN) was selected as the internal standard to determine the TG content, because NNN was not present in edible oils. The relative response factors (RRS) were calculated for all the analyzed standards of TGs, obtained by relating the area and concentration of each standard to the concentration and area of the IS.

### 3.6. Identification of TG

In line with Pacheco et al. (2014) and AOCS Official Method Ce 5b-89 (2009), Equation (1) was used to calculate the relative retention time, which relates the retention time (rt) of an analyte to solvent and IS retention time.

(1) $$\mathrm{rrt}=\frac{{\mathrm{rt}}_{\mathrm{analyte}}-{\mathrm{rt}}_{\mathrm{solvent}}}{{\mathrm{rt}}_{\mathrm{IS}}-{\mathrm{rt}}_{\mathrm{solvent}}}$$

rrt: Relative retention time; rt_analyte_: Retention time of TAGs; rt_solvent_: Retention time of dichloromethane; rt_IS_: Retention time of internal standard.

## 4. Conclusions

This work has shown that the HTGC-FID method can be used to identify blending of rapeseed oil with olive oil at the level of 1%. The method described is a fast one-step method (28 min) involving only dilution of the oil in a solvent, and it allows separation and quantitation of 20 individual TGs. For TGs typical of edible oils, the method’s recovery, precision, and repeatability provide reliable indications of oil blending. When olive oil is added to rapeseed oil, or when olive oil is adulterated with rapeseed oil at the level of 1%, this method can be used for quality control of these oils. Three triacylglycerols were identified as indicators of the addition of olive oil to rapeseed oil (PPO, PPL, PSO). The presence of OOLn indicated the adulteration of olive oil with refined rapeseed oil.

## Figures and Tables

**Table 1:** Chemical and validation parameters used for the analysis of TG by GC-FID method.

TG	CN	DB	ECN	rrt	RRF	Recovery (%)	LOD (µg/mL)	LOQ (µg/mL)	Precision (%)	Repeatability (%)	RSD (%)
LLL	54	6	42	0.97	0.44	21	0.001	0.001	2.8	8.0	1.80
LLO	54	5	44	0.95	1.47	148	0.083	0.251	20.0	52.6	3.53
LOL	54	5	44	0.95	0.73	74	0.169	0.511	7.0	19.5	2.63
OOLn	54	5	44	0.96	0.42	36	0.330	1.000	17.7	49.6	11.74
OOL	54	4	46	0.94	1.11	56	0.109	0.330	18.0	50.3	4.46
LLS	54	4	46	0.95	0.85	41	0.140	0.424	2.26	6.3	0.73
PPL	50	2	46	0.79	1.11	73	0.125	0.379	7.3	20.7	1.84
PPP	48	0	48	0.70	0.86	79	0.140	0.421	2.2	6.3	0.73
OOP	52	2	48	0.85	1.39	140	0.088	0.267	0.3	0.7	3.64
OPO	52	2	48	0.86	1.24	125	0.181	0.549	0.5	1.5	2.87
OOO	54	3	48	0.93	2.28	102	0.053	0.16	26.7	74.8	3.23
PPS	50	0	50	0.77	1.72	108	0.001	0.001	20.7	57.9	3.33
PSO	52	1	50	0.85	1.52	74	0.170	0.515	22.6	63.4	4.11
OOS	54	2	50	0.92	1.72	99	0.067	0.202	0.2	0.6	0.57
SSO	54	1	52	0.91	1.00	61	0.001	0.001	8.2	23.0	2.27
SSS	54	0	54	0.91	1.00	90	0.010	0.010	2.1	20.1	1.92
NNN	57	0	57	1.00	1.00	100	0.117	0.354	16.1	45.1	4.45

CN: Carbon numbers; DB: Double bond numbers; ECN: Equivalent of carbon number; RRF: Relative response factor; rrt: Relative retention time. Repeatability is expressed as the relative standard deviation of TGs; RSD: Relative standard deviation; LOD: Limits of detection; LOQ: Limits of quantitation.

**Table 2:** Triacylglycerol levels in refined rapeseed oil, olive oil, and their blends (mg/g).

TGs	RRO	OO	Blended Oils OO/RRO				Blended Oils RRO/OO			
			1.0%	2.5%	5.0%	10.0%	1.0%	2.5%	5.0%	10.0%
PPO	Nd	20.8 ± 2.8	0.8 ± 0.3	3.9 ± 0.8	4.3 ± 0.3	7.9 ± 0.3	18.4 ± 0.8	17.0 ± 0.5	15.0 ± 0.8	12.4 ± 0.6
PPL	Nd	9.3 ± 4.1	0.9 ± 0.2	1.9 ± 0.6	2.1 ± 0.1	2.7 ± 0.1	8.5 ± 0.4	8.4 ± 0.3	8.1 ± 0.5	7.4 ± 0.4
PSO	Nd	6.7 ± 0.8	0.2 ± 0.1	1.4 ± 0.5	1.9 ± 0.1	5.8 ± 0.3	7.6 ± 0.3	6.0 ± 0.4	4.1 ± 0.5	3.9 ± 0.2
OOP	47.4 ± 2.1	254.2 ± 8.2	71.5 ± 3.7	82.4 ± 2.3	105.7 ± 4.3	124.7 ± 5.6	246.5 ± 8.7	235.0 ± 7.1	212.0 ± 9.6	190.9 ± 10.1
POL	53.3 ± 2.6	60.6 ± 2.5	48.3 ± 2.3	50.1 ± 2.0	54.2 ± 3.0	42.4 ± 2.0	55.2 ± 2.7	49.0 ± 2.3	43.3 ± 2.1	43.7 ± 1.9
POLn	26.9 ± 1.4	1.6 ± 0.5	26.1 ± 1.7	24.3 ± 1.6	23.0 ± 1.1	18.9 ± 0.9	2.8 ± 0.1	4.9 ± 0.2	6.4 ± 0.2	11.7 ± 0.3
OOS	13.7 ± 4.1	33.3 ± 1.1	11.7 ± 0.8	14.5 ± 0.8	20.2 ± 1.4	23.3 ± 1.1	33.2 ± 1.4	31.7 ± 1.8	29.7 ± 1.2	22.5 ± 0.9
OOO	424.1 ± 10.1	516.5 ± 11.1	429.3 ± 9.6	430.2 ± 9.3	443.8 ± 8.6	470.0 ± 10.8	515.6 ± 8.8	507.8 ± 9.8	504.9 ± 10.6	500.2 ± 9.9
OOL	194.6 ± 8.3	88.5 ± 6.4	183.3 ± 7.7	155.4 ± 7.5	137.7 ± 5.3	121.5 ± 6.2	83.6 ± 5.7	91.0 ± 4.5	107.6 ± 5.2	114.3 ± 4.6
OOLn	117.8 ± 5.7	Nd	116.8 ± 5.1	97.3 ± 3.8	80.8 ± 2.1	68.2 ± 3.1	2.2 ± 0.1	4.1 ± 0.3	6.0 ± 0.4	12.2 ± 0.6
LLO	34.9 ± 2.2	8.4 ± 0.6	32.6 ± 0.9	30.7 ± 1.1	28.3 ± 1.1	24.9 ± 0.9	8.7 ± 1.1	9.4 ± 0.5	9.9 ± 0.5	12.7 ± 0.6

RRO: Refined rapeseed oil; OO: Olive oil.

## Abbreviations

Triacylglycerols (TG); monoacylglycerols (MAG); diacylglycerols (DAG);1,2-linoleoyl-3-oleoyl-*sn*-glycerol (OLL); 1,3-palmitoyl-2-oleoyl-*sn*-glycerol (POP); 1,2-palmitoyl-3-oleoyl-*sn*-glycerol (PPO), 1,3-palmitoyl-2-linoleoyl-*sn*-glycerol (PLP); trinonadecanoyl-glycerol(NNN); trioleoyl-glycerol (OOO); 1,2-oleoyl-3-*sn*-palmitoyl-glycerol (OOP); 1,2-palmitoyl-3-linolein-*sn*-glycerol (PPL); 1,3-oleoyl-2-palmitoyl-*sn*-glycerol (OPO); 1,2-palmitoyl-3-stearoyl-*sn*-glycerol (PPS); 1,2-oleoyl-3-stearoyl-*sn*-glycerol (OOS); 1,2-stearoyl-3-oleoyl-*sn*-glycerol (SSO); 1-palmitoyl-2-stearoyl-3-oleoyl-*sn*-glycerol (PSO); 1,2-oleoyl-3-linoleoyl-*sn*-glycerol (OOL); 1,2-linoleoyl-3-oleoyl-*sn*-glycerol (LLO); 1,2-oleoyl-3-linolenoyl-*sn*-glycerol (OOLn); 1,3-linoleoyl-2-oleoyl-*sn*-glycerol (LOL); tristearoyl-glycerol (SSS); tripalmitoyl-glycerol (PPP); 1,2-linoleoyl-3-steareroyl-*sn*-glycerol (LLS); High Temperature Gas Chromatography- Flame Ionization Detector (HTGC-FID).
